# Compounding tropical and stratospheric forcing of the record low Antarctic sea-ice in 2016

**DOI:** 10.1038/s41467-018-07689-7

**Published:** 2019-01-02

**Authors:** Guomin Wang, Harry H. Hendon, Julie M. Arblaster, Eun-Pa Lim, S. Abhik, Peter van Rensch

**Affiliations:** 1 Bureau of Meteorology, Docklands 3008, VIC Australia; 2 ARC Centre of Excellence for Climate Extremes, Monash University, Clayton 3800, VIC Australia; 3 School of Earth, Atmosphere and Environment, Monash University, Clayton 3800, VIC Australia; 4 National Center for Atmospheric Research, Boulder, CO 80301 USA

## Abstract

After exhibiting an upward trend since 1979, Antarctic sea ice extent (SIE) declined dramatically during austral spring 2016, reaching a record low by December 2016. Here we show that a combination of atmospheric and oceanic phenomena played primary roles for this decline. The anomalous atmospheric circulation was initially driven by record strength tropical convection over the Indian and western Pacific Oceans, which resulted in a wave-3 circulation pattern around Antarctica that acted to reduce SIE in the Indian Ocean, Ross and Bellingshausen Sea sectors. Subsequently, the polar stratospheric vortex weakened significantly, resulting in record weakening of the circumpolar surface westerlies that acted to decrease SIE in the Indian Ocean and Pacific Ocean sectors. These processes appear to reflect unusual internal atmosphere-ocean variability. However, the warming trend of the tropical Indian Ocean, which may partly stem from anthropogenic forcing, may have contributed to the severity of the 2016 SIE decline.

## Abstract (web summary)

Antarctic sea ice extent declined dramatically in austral spring 2016. This study shows the decline was initially driven by tropical convection resulting in a wave-3 circulation pattern, followed by weakened circumpolar surface westerlies initialised in the polar stratospheric vortex.

## Introduction

Sea-ice extent (SIE) over the two poles shows contrasting trends based on satellite observations since 1979. Arctic SIE has decreased substantially^1^ whereas Antarctic SIE has exhibited a small but significant increase^2^. Climate models, when forced with all major natural and anthropogenic forcings over the same period, simulate SIE reduction in both the Arctic and Antarctic regions, inconsistent with the observed Antarctic SIE increase^3^. Several mechanisms have been suggested for the weak positive Antarctic SIE trend, such as internal variability^4,5^, decadal modulation by the Interdecadal Pacific Oscillation^6,7^, cooling of the Southern Ocean surface due to the observed strengthening of the westerly winds and positive trend in the Southern Annular Mode (SAM) resulting from ozone depletion^8,9^ and freshening of the ocean surface thus acting to reduce the upwelling of warmer subsurface waters^10–12^. However, there is no consensus as to the cause of the upward trend.

On shorter time scales, internal modes of variability such as El Niño/Southern Oscillation (ENSO) and SAM have been identified for having played important roles in influencing seasonal variations of SIE^13–15^. The primary processes for these climate modes to influence SIE are through induced changes in atmospheric heat transport and sea-ice drift resulting from changes of the atmospheric circulation around Antarctica. The circulation anomalies often manifest in a zonally symmetric pattern in response to SAM or wave like structures in response to ENSO. The influence of ENSO on Antarctic sea-ice is primarily through the atmospheric Rossby wave trains forced by anomalous tropical convection that accompanies anomalous sea surface temperature (SST) in the tropical Pacific^14,16^. ENSO strongly co-varies with the Indian Ocean dipole (IOD) mode in austral spring September-November, which also drives a Rossby wave train that emanates into higher latitudes of the Southern Hemisphere (SH)^17^ and can also influence SIE^18^.

**Fig. 1:**
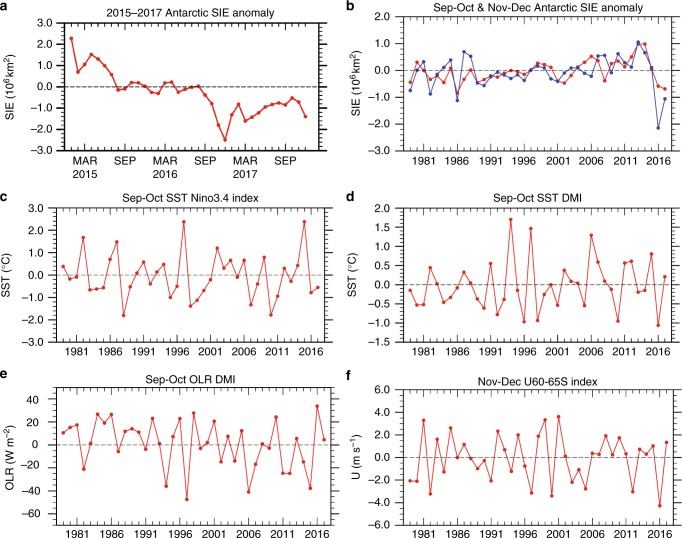
Observed indices of Antarctic sea-ice and several key climate drivers during austral spring 1979–2017. **a** Monthly anomaly during 2015–2017 of Antarctic sea-ice extent (SIE) (10^6^ km^2^). The same time series for full period 1979–2017 is shown in Supplementary Figure 1. **b** SIE anomaly (10^6^ km^2^) for September–October (Sep–Oct, red curve) and November–December (Nov–Dec, blue curve); **c** Sep–Oct SST Niño3.4 index (°C); **d** Sep–Oct Indian Ocean Dipole Mode index for SST, denoted as SST Dipole Mode Index (SST-DMI, °C); **e** same as **d** but the index is calculated using the anomalous OLR, denoted as OLR-DMI (W m^−2^); **f** Nov–Dec zonal wind index U_60–65S_ (m s^−1^). All anomalies are deviations relative to 1979–2015 means. Other details see Methods. Plots were generated using the NCAR Command Language version 6.4.0 (www.ncl.ucar.edu)

Antarctic SIE began a rapid decline from September 2016^19^ (Fig. 1a) resulting in record low anomalies by late austral spring, which then partially recovered back to near normal by austral autumn 2017. The unprecedented decline in SIE during spring 2016 raises the following question: What were the processes that promoted the decline? Was the decline a temporary shift or the beginning of a long-term downward trend as predicted by the climate models? Was the cause natural variability or a response to increasing greenhouse gases? Several studies^19,20^ have highlighted the role played by atmospheric circulation anomalies for driving the SIE decline in 2016. During September–October (Sep–Oct) the atmospheric circulation exhibited a wave number 3 pattern in the Southern Hemisphere extratropics, that acted to decrease sea-ice regionally where the anomalous surface flow was toward the pole^19,21^. The circumpolar westerlies then weakened more zonally uniformly during November–December (Nov–Dec) indicative of a strong low SAM pattern, which acted to increase warm Ekman transport toward the pole^19,20^.

Here we investigate the causes of these unusual circulation patterns during September–December 2016 that resulted in record low SIE by the end of 2016. We are especially interested in discerning the roles of internal atmosphere-ocean variability and the possible contribution from global warming. Because of the development of two distinctive circulation patterns during this extended spring season, we discuss Sep–Oct and Nov–Dec periods sequentially^20^. The Sep–Oct and Nov–Dec SIE anomalies since 1979 are shown in Fig. 1b. The SIE in Sep–Oct 2016 (red line) was the third lowest (−0.59 × 10^6^ km^2^) since 1979, slightly weaker than the record low in 1986 (−0.84 × 10^6^ km^2^). The SIE in Nov–Dec 2016 (blue line) was a record low (−2.14 × 10^6^ km^2^), far stronger than the second lowest in 1986 (−1.12 × 10^6^ km^2^). These record low and near-record low values in 2016 are in contrast with the record high values that occurred in 2013.

While previous studies have emphasised the persistent warming of the polar Southern Ocean from the El Niño of 2015^20^(Fig. 1c) as an important driver for this record sea ice decline, here we introduce the key role played by anomalous conditions in the tropical Indian Ocean (IO) and western Pacific associated with a record negative IOD event during austral spring 2016^22,23^ (Fig. 1d) that promoted the prominent wave-3 pattern during Sep–Oct at high latitudes. The subsequent record weakening of the circumpolar surface westerlies and shift to low SAM (Fig. 1f) during Nov–Dec emerged after the tropical Madden Julian Oscillation in early November acted to curtail the IOD-forced convective anomalies and associated wave-3 circulation pattern and a dramatic weakening of the stratospheric polar vortex beginning in October then extended downward to the surface during Nov–Dec.

## Results

### Rossby wave train during September–October 2016

**Fig. 2:**
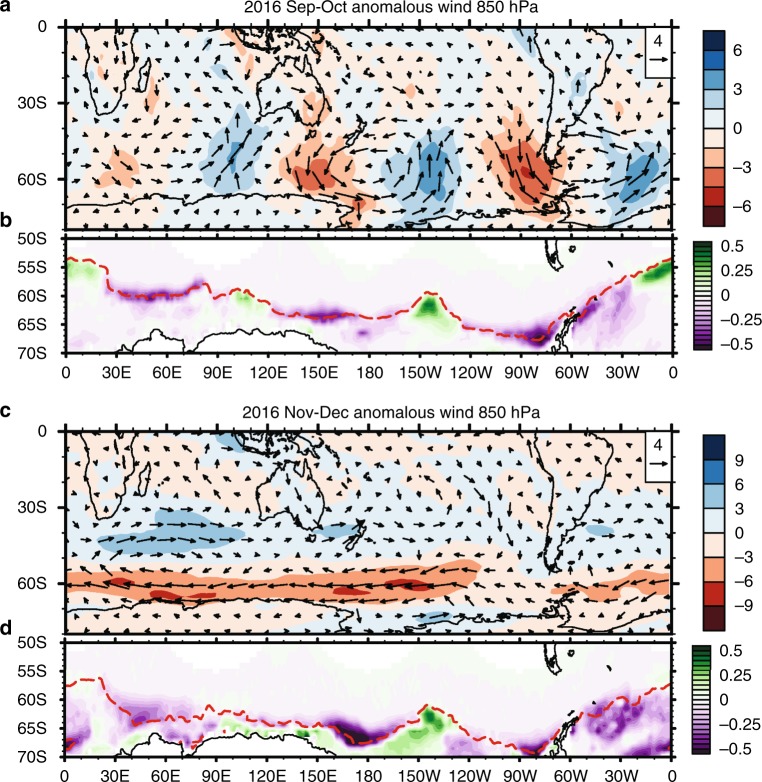
Atmospheric circulation and sea-ice concentration during September to December 2016. **a** Sep–Oct 850 hPa wind anomaly (vectors, scale in upper right, m s^−1^); Shading is meridional wind component with northerly coloured in reds and southerly coloured in blues (interval 1.5 m s^−1^); **b** Sep–Oct Antarctic sea-ice concentration (SIC) anomaly (shading; interval 0.05); the 0.15 contour is highlighted with red dashed curve. For clarity, SIC anomaly is only displayed in the latitudes poleward of 50**°**S. **c**, **d** are the same as **a**, **b**, respectively, but for Nov–Dec 2016. The shading in **c** is zonal wind component with easterly coloured in reds and westerly coloured in blues (interval 3 m s^−1^). Plots were generated using the NCAR Command Language version 6.4.0 (www.ncl.ucar.edu)

The lower level atmospheric circulation anomalies during Sep–Oct 2016 appear as a wave train arcing across the mid-high latitudes (Fig. 2a), with a close correspondence between the variations in Antarctic sea-ice, represented by sea-ice concentration (SIC) (Fig. 2b), and the meridional wind anomalies (shading in Fig. 2a). SIC decreases in conjunction with anomalous northerlies and increases in conjunction with southerlies through associated anomalous poleward heat advection and sea ice drift. SIC anomalies were negative over the west Pacific to west Ross Sea sector, Bellingshausen and Amundsen Seas sector, and to lesser extent over the western Indian Ocean sector^19^.

Rossby wave theory suggests that changes in tropical convection and corresponding anomalous diabatic heating can excite Rossby wave trains that teleconnect the region of tropical forcing to the extratropics^24^. In the SH, the Rossby wave response to tropical convective forcing manifests as a stationary wave train propagating poleward and eastward from the source, thereby resulting in persistent circulation anomalies that can influence surface thermal conditions and sea-ice across most sectors surrounding the Antarctic^25,26^.

**Fig. 3:**
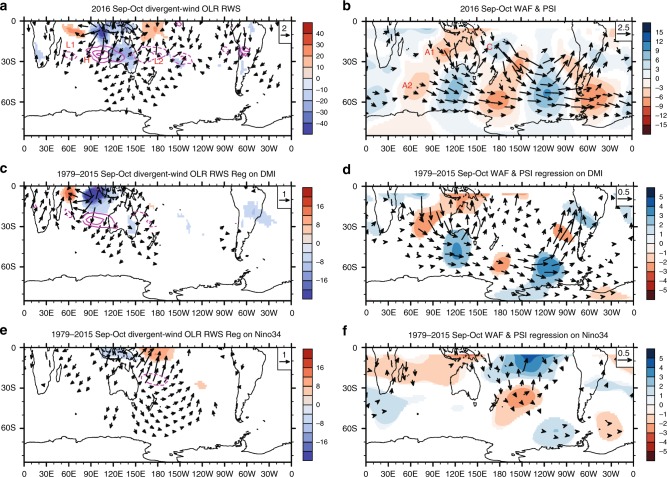
Rossby wave train and source during September–October 2016. **a** 200 hPa divergent wind anomaly (vectors, scale in upper right, m s^−1^), Rossby wave source (RWS, magenta contours, positive (anticyclonic) solid and negative (cyclonic) dashed, zero line omitted; interval 4 × 10^−11^s^−2^, see Methods) and outgoing longwave radiation (OLR) anomaly (shading; interval 4 Wm^−2^) for Sep–Oct 2016. **b** 200 hPa eddy streamfunction anomaly (shading; interval 3 × 10^6^ m^2^s) and anomalous wave activity flux (WAF, vectors, m^2^ s^−2^) for Sep–Oct 2016. **c**–**f** are same as **a**, **b** but displayed fields are derived by multiple linear regression onto the DMI index (**c**, **d**) and Nino34 SST index (**e**, **f**) using historical data for the period 1979–2015 (see Methods). The anomalies in **c**, **d** and **e**, **f** are scaled by the observed magnitude of the DMI and Nino34 index, respectively, in Sep–Oct 2016. Contouring/shading is as in **a** and **b**, except RWS interval is 2 × 10^−11^ s^−2^, and eddy streamfunction interval is 1 × 10^6^ m^2^s. Anomalies and regression coefficients are shown only for *p* < 0.10 using two-sided *t*-test. For clarity, divergent wind weaker than 0.8 ms^−1^ in **a**, and weaker than 0.3 ms^−1^ in **c** and **e**, WAF weaker than 0.8 m^2^s^−2^ in **b**, and weaker than 0.1 m^2^s^−2^ in **d** and **f** are masked. In the Southern Hemisphere, negative streamfunction anomaly corresponds to positive geopotential height anomaly and thus is coloured in reds; similarly, positive streamfunction anomaly is coloured in blues. Plots were generated using the NCAR Command Language version 6.4.0 (www.ncl.ucar.edu)

During Sep–Oct 2016, weak La Niña conditions were developing in the Pacific (Fig. 1c), while a record strong negative IOD had reached its mature stage in the tropical Indian Ocean as reflected by the IOD Mode Index (DMI)^22,23^ (Fig. 1d). The anomalous SST patterns associated with this La Niña and negative IOD favoured a tri-pole pattern of suppressed-enhanced-suppressed convection in western Indian Ocean through to the western Pacific Oceans (Fig. 3a, shading). An index representing this tropical convective anomaly (Methods) exhibited a record value in Sep–Oct 2016 (Fig. 1e).

These record anomalies in tropical convection forced anomalous divergent outflow and inflow in the upper troposphere (Fig. 3a, vectors), which act as an effective Rossby wave source (RWS; Methods) in the subtropics by anomalously advecting the mean meridional gradient of absolute vorticity that maximises along about 25°S^27,28^. An anticyclonic RWS emerges off the west coast of Australia in association with the southward divergent flow from the enhanced convection in the eastern Indian Ocean (purple contours lettered “H” in Fig. 3a). Similarly, the anomalous northward divergent in-flow driven by suppressed convection in the western Indian Ocean and more pronouncedly in the western Pacific produced cyclonic RWS centred approximately at 65°E (“L1”) and 165°E (“L2”), respectively.

The anticyclonic RWS in the eastern Indian Ocean (“H” in Fig. 3a) appears to drive an anticyclonic streamfunction anomaly off the northwest coast of Australia (“A1” in Fig. 3b), and the cyclonic RWS off the northeast coast of Australia (“L2” in Fig. 3a) drives a cyclonic streamfunction anomaly (“C” in Fig. 3b). These subtropical streamfunction anomalies are then associated with wave trains that arc poleward and eastward into the SH higher latitudes. Wave activity flux anomalies (vectors in Fig. 3b; Methods), which track the group velocity of the Rossby wave train, indicate that the RWS in the eastern Indian Ocean is the source of the wave train that arcs poleward to the west of Australia, and then eastward across the Southern Ocean along the waveguide of the high latitude westerly jet^27^. The cyclonic RWS in the western Pacific is similarly associated with a wave train that propagates poleward and eastward in the western Pacific, avoiding the region around New Zealand where meridional gradient of absolute vorticity becomes negative (Supplementary Figure 2) and so prohibits Rossby wave propagation^29,30^. This wave train perhaps is partially refracted back to the tropics in the eastern Pacific (indicated by the equatorward pointing wave activity flux vectors). However, the two wave trains appear to constructively add to the cyclonic-anticyclonic anomalies to the southwest and southeast of South America, thus contributing to the zonal wavenumber 3 pattern along 60°S. Divergence of wave activity flux in the southern Indian Ocean indicates an additional anticyclonic source of the extratropical Rossby wave train (labelled “A2” in Fig. 3b). This additional wave source has previously been shown to typically develop during negative IOD years, resulting from feedback of altered transient eddies in the high latitude storm track onto the seasonal mean anomalous circulation^27^.

The extratropical circulation anomalies are similarly signed throughout the depth of the troposphere (i.e. compare Fig. 3b with the implied circulation centres near the surface from Fig. 2a), consistent with theoretical considerations for forcing of extratropical circulation anomalies by tropical convection^24^, and so are indicative of the anomalous winds at the surface that would directly impact sea ice.

The role played by IOD and ENSO in promoting the Rossby wave trains that affected SIE during Sep–Oct 2016 is clarified by multiple regression of winds, outgoing longwave radiation (OLR) and streamfunction onto indices of the IOD (Fig. 3 middle panels) and ENSO (Fig. 3 bottom panels) using the historical record 1979–2015. For display, the resulting regressions are scaled by the magnitude of the IOD and ENSO indices in 2016 (Methods). This method successfully isolates the anomalous convection, RWS, and wave train emanating from the tropical Indian Ocean associated with the IOD and from the tropical Pacific associated with ENSO. During 2016, the wave train emanating from the Indian Ocean has stronger amplitude and spans both the eastern and western hemispheres compared to the wave train emanating from the Pacific associated with La Niña. The sum of these two patterns explains about half of the high latitude anomalies observed during Sep–Oct 2016 (figure not shown). The other half plausibly comes from internal variability that cannot be described by the simple regression model.

The analysis presented in Fig. 3 indicates that the record negative IOD played a key role in generating the wave-3 pattern at high latitudes that affected SIE around the breadth of Antarctica during Sep–Oct 2016. The weak La Nina conditions in the Pacific played a more secondary role by contributing constructively to the anomalous circulation to the south of South America. The stronger forcing from the tropical Indian Ocean is supported by comparable regression results using reforecasts from a coupled model (Supplementary Figure 3). The results are also consistent with that obtained with an atmosphere-only model experiment with a specified convective heating representing precipitation anomaly in September–November 2016 in the eastern Indian/western Pacific Ocean^31^.

### Low SAM circulation during November–December 2016

**Fig. 4:**
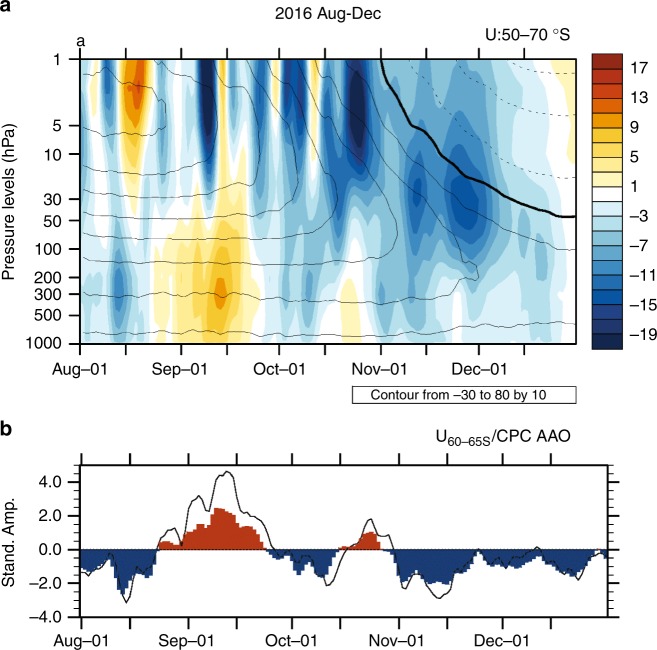
Daily anomalies of Antarctic circulation in August to December 2016. **a** Zonal mean zonal wind anomalies averaged 50–70˚S (shading, m s^−1^) superimposed with climatological zonal mean zonal wind (contour interval 10 m s^−1^, easterly values dashed, and zero contour thickened). **b** Standardised amplitudes of the daily U_60–65S_ index (shading) and the NOAA CPC Antarctic Oscillation index that represents SAM (curve). Plots were generated using the NCAR Command Language version 6.4.0 (www.ncl.ucar.edu)

Although a significant decline of the SIE occurred in Sep–Oct 2016, the most drastic decline occurred during Nov–Dec 2016 (Fig. 1a). This rapid decline was associated with development of a low SAM (Fig. 4b)^19,20^, which has been shown to favour sea-ice loss through Ekman transport^32^ and surface temperature - sea ice interaction^21,33^. While the strength of low SAM was moderate, the associated easterly anomalies along the periphery of the sea-ice (60–65°S) was record strong in Nov–Dec since 1979 (Fig. 1f).

The low SAM of late 2016 was unexpected because La Niña usually acts to promote high SAM^20,34,35^. So why did a strong low SAM develop during Nov–Dec 2016 when La Niña conditions persisted? We argue that this low SAM was driven by the combined effects of the downward coupling from the polar stratosphere due to a significant weakening of the polar vortex during October–December 2016 and the passing of an Madden Julian Oscillation (MJO) during November 2016.

Stratospheric polar vortex variations and their downward coupling to the surface are a key driver of variations of the SAM in austral spring to summer^36–38^. In early spring 2016, anomalous upward propagation of planetary wave activity from the upper troposphere into the stratosphere weakened the polar vortex in the upper stratosphere substantially^39^. This weakened vortex propagated downward to the surface (Fig. 4a), promoting low SAM and weakened circumpolar surface westerlies during Nov–Dec (Fig. 4b). This weakening of the polar vortex is viewed as an earlier than normal breakdown of the vortex that typically occurs in late spring (compare the anomalous zonal winds to the climatological zonal winds in Fig. 4a). This coupling of the weakened surface circumpolar westerlies during Nov–Dec to the preceding weakening of the stratospheric polar vortex during October is confirmed in the historical record (Supplementary Figure 4a) and is also supported in reforecasts with a coupled model (Supplementary Figure 4b).

We note in late October–November 2016 a higher concentration of the polar stratospheric ozone with a substantially smaller ozone hole was observed (http://www.cpc.ncep.noaa.gov/products/stratosphere/sbuv2to/gif_files/ozone_hole_plot.png). The enhanced polar stratospheric ozone in these months can also be associated with low SAM in subsequent months^40^, with a strong coupling between the polar vortex and ozone anomalies.

During early November 2016, a strong MJO traversed the IO that acted to suppress convection in the eastern IO and enhance convection over the western Pacific (Supplementary Figure 5). This MJO event thus acted to rapidly weaken the SST anomalies (Supplementary Figure 6a) and tropical convective anomalies (Supplementary Figure 6b) associated with the negative IOD^22^, which were the source of the wave-3 pattern during Sep–Oct 2016. Thus the MJO helped to enable the more zonally uniform weakening of the circumpolar surface westerlies, in response to the earlier than normal breakdown of the polar stratospheric vortex, to emerge in Nov–Dec 2016.

Though the circulation anomalies associated with SAM are predominantly annular, its impact on sea-ice is non-annular^41,42^ with preferred regions of influence in the west Antarctic^43^ and IO sector^44^. Once low SAM was established in Nov 2016, SIE further decreased in the IO, Pacific, and Weddell Sea sectors (Fig. 2d; Supplementary Figure 7).

**Fig. 5:**
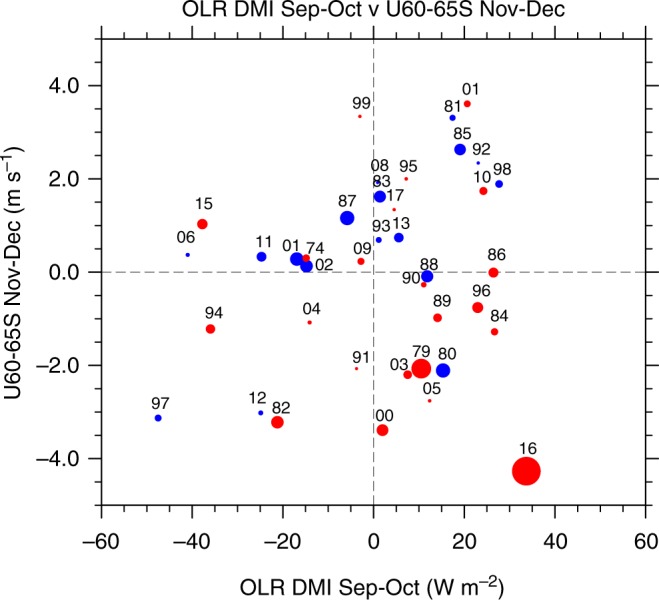
Scatter plot of OLR-DMI in Sep–Oct and U_60–65S_ in Nov–Dec during 1979–2017. The data used here are the same as shown in Fig. 1e, f. Each dot is sized and coloured according to magnitude and sign of the SIE change from winter to spring (i.e., September–December mean minus June–August mean; red for SIE decrease and blue for SIE increase). The dot sizes are proportional to the magnitude of the SIE change. Year is shown by the last two digits above each data point. Plots were generated using the NCAR Command Language version 6.4.0 (www.ncl.ucar.edu)

Our study suggests that the record low sea-ice concentration during September–December 2016, which previously has been primarily attributed to anomalous atmospheric circulation^20,21^, was largely a result of the sequential occurrence of tropical and stratospheric forcing. This included record strength negative IOD during Sep–Oct followed by an unusually early breakdown of the stratospheric polar vortex which resulted in record weakening of the surface circumpolar westerlies during Nov–Dec. Although the strength of the negative IOD and the weakened circumpolar westerlies are not well separated individually from their previous records (Fig. 1e, f), their sequential combination during Sept–Oct and Nov–Dec 2016 is clearly an outlier (Fig. 5).

Our results also suggest the record decline of SIE at the end of 2016 was largely the result of naturally occurring variability. This conclusion is bolstered by the subsequent return to near normal SIE by mid 2018 (Fig. 1a). However, we cannot exclude a possible role of anthropogenic forcing. For instance, ongoing warming of the tropical IO in austral autumn and winter^45^ appears to have contributed to the formation of the extreme negative IOD in 2016^23^. Anthropogenic forcing is considered a major contributor to this warming trend^46^. Thus, anthropogenic forcing may have contributed to the record IOD convective anomalies that developed during 2016. Furthermore, the chance of low SAM events like that in Nov–Dec 2016 are tightly tied to warming events in the polar stratosphere which may become more likely in the future as a result of the mending of the Antarctic ozone hole. Ozone depletion and the increase of the greenhouse gases have both contributed to the recent trend towards high SAM in austral summer^47^. As the Antarctic ozone hole begins its recovery^48,49^, more frequent occurrence of low SAM might be expected compared to the past 40 years.

## Methods

### Data sets and indices

Monthly mean sea-ice concentration data sets are from the U.S. National Snow and Ice Data Center (NSIDC)(Swift and Cavalieri^50^) using the NASA Team algorithm. The monthly mean winds, geopotential height, and mean sea level pressure are from ERA-Interim. Outgoing longwave radiation (OLR) data is obtained from twice-daily observations interpolated in time and space and then averaged to monthly means^51^. The original data set has been updated for 2013 onwards using the same method (M Wheeler, personal communication). We have used monthly SST data from NOAA optimally interpolated (OI) version 2 (NOAA-OIv2)^52^, and HadISST^53^. We only show results based on NOAA-OIv2 in this paper as the results are consistent from both datasets.

We regridded the above data sets to a common 1.5° × 1.5° latitude–longitude grid before diagnosing, and used 1979–2015 mean as reference for deriving anomalies.

The sea-ice extent (SIE) is defined as the sum of areas covered by grids whose sea ice concentration is at least 15%.

We use SST Niño 3.4 (averaged SST anomaly over 170°W–120°W, 5°N–5°S) to represent ENSO and name it as SST-Nino3.4. The Indian Ocean Dipole (IOD) mode index (DMI) for SST is calculated as the anomalous SST difference between the western equatorial Indian Ocean box (50°E–70°E and 10°S–10°N) and the eastern equatorial Indian Ocean box (90°E–110°E and 10°S–0°N). We name it as SST-DMI. To represent the strength of convective activity in the Indian Ocean we introduce a new index in the same manner as the SST-DMI but using OLR as input. We name this index as OLR-DMI. The SST-DMI and OLR-DMI are highly anti-correlated (*r* = −0.9, *p* < 0.01): a warm SST anomaly at the eastern equatorial Indian Ocean corresponds to a strong convection in the same region.

Daily and monthly Antarctic Oscillation (AAO, we use AAO and SAM interchangeably) indices are obtained from NOAA Climate Prediction Center (CPC). They are constructed by projecting the daily and monthly mean NCEP-NCAR reanalysis^54^ 700-hPa height anomalies onto the leading EOF mode, and are normalised by the standard deviation of the monthly index (1979–2000 base period). For better representing SAM impact on Antarctic sea-ice, an alternative zonal wind index is introduced. This index is defined as the zonal and meridional averaged zonal wind at 850 hPa. The meridional average is from 60°S to 65°S. We choose this latitudinal band because the annual and zonal average of the Antarctic sea-ice edge, defined as the equatorward position of 15% contour of SIC, is near 65°S. We name this index as U_60–65S_. The correlation *r* between monthly U_60–65S_ and the CPC SAM index over 1979–2015 ranges from 0.89–0.91 for different seasons (for Nov–Dec *r* = 0.91, *p* < 0.01).

### Rossby wave source, wave activity flux and wave numbers

An enhanced tropical convection anomaly induces vertical motion anomaly and the associated lower-level convergence and upper-level divergence anomaly, which produce an anomalous vorticity source in the tropics. The upper level component of this vorticity source, denoted the Rossby wave source (RWS)^55^ and can be defined as

1 $${\rm{RWS}} = - {\mathbf{v}}_{\mathrm{\chi }} \cdot \nabla \zeta - \zeta D \approx - {\mathbf{v}}_{\mathrm{\chi }}^\prime \cdot \nabla \bar \zeta - \bar \zeta D^\prime$$

where *ζ* is the 2D absolute vorticity, **v**_χ_ is the divergent component of the horizontal wind vector and *D* is the horizontal wind divergence, and the overbar and prime represent the climatological mean and perturbation, respectively. The RWS are well approximated by their linearised components S1 ($- {\mathbf{v}}_{{\chi }}^\prime \cdot \nabla \bar \zeta$), the advection of mean absolute vorticity by anomalous divergent flow, and S2 ($- \bar \zeta D^\prime$), the vortex stretching term, or the generation of vorticity by anomalous divergence^56^. Here we only show S1, as this component is more effective at exciting extratropical teleconnections from the tropical heating^55^.

Rossby wave activity flux^57^ is used to investigate the Rossby wave propagation from the tropics. This diagnostic tool illustrates the evolution and propagation of stationary Rossby wave disturbances on a zonally varying basic flow. For slowly varying basic flows, the wave activity flux **W** is parallel to the local group velocity. Here we focus on horizontal component of **W** on 200 hPa:

2 $${\mathbf{W}} = \frac{1}{{2\left| {{\bar{\boldsymbol U}}} \right|}}\left[ {\begin{array}{*{20}{c}} {\bar u\left( {\psi _x^{\prime 2} - \psi ^{\prime} \psi _{xx}^{\prime} } \right) + \bar v\left( {\psi _x^{\prime} \psi _y^\prime - \psi ^\prime \psi _{xy}^{\prime 2}} \right)} \\ {\bar u\left( {\psi _x^{\prime} \psi _y^{\prime} - \psi ^{\prime} \psi _{xy}^{\prime} } \right) + \bar v\left( {\psi _y^{\prime 2} - \psi ^{\prime} \psi _{yy}^{\prime 2}} \right)} \end{array}} \right]$$

where ${\bar{\boldsymbol U}} = (\bar u,\bar v)$ is the basic wind field; $\psi ^\prime$ is the streamfunction anomaly derived from geopotential height through the use of quasigeostrophic approximation. The regression pattern of **W** shown in Fig. 3d, f are calculated using the regressed Sep–Oct mean geopotential height anomalies as input in (2). The basic flow used is the Sep–Oct mean over 1979–2015.

Rossby wave propagation from the tropics to the extratropics is modulated by the mean zonal flow. We use the Rossby wave tracing theory^24,29^ to illustrate the impact of the mean flow on the stationary wave paths. From the Rossby wave dispersion relation in the linearised barotropic vorticity equation^24^, we obtain the total stationary Rossby wavenumber ${\boldsymbol K}_{\mathrm{s}}$:

3 $${\boldsymbol K}_{\mathrm{s}} = (\frac{{\beta ^ \ast }}{\bar {\boldsymbol{U}}})^{1/2}$$

where $\bar U$ is the mean zonal flow and

4 $$\beta ^ \ast = \beta - \frac{{\partial ^2\bar {\boldsymbol U}}}{{\partial y^2}}$$

is the meridional gradient of the mean absolute vorticity and *β* = ∂*f*/∂*y* is the variation of the Coriolis parameter with latitude. From Eq. (3) the total wavenumber will be real when $\beta ^ \ast$ is positive and the mean flow is westerly. When the absolute vorticity gradient goes to zero or negative, Rossby wave propagation is not supported and the ray paths will tend to be reflected before reaching these regions^29^.

### Reforecasts data

The reforecasts used in supporting the observational results were from Australian Community Climate and Earth System Simulator Seasonal Prediction System-1 (ACCESS-S1)^58^. The ACCESS-S1 is based on the UK Met Office’s global coupled model seasonal forecast system GloSea5-GC2^59^ with some enhancements to the ensemble generation strategy. Reforecasts have been conducted for start dates over 1990–2012 with 11 ensemble members. We have used reforecasts initialized on 01 September and extending through December here.

The bias corrected reforecast anomaly is formed by removing the reforecast climatology from individual reforecasts. The linear regression is then applied to the Sep–Oct mean reforecast anomaly (Supplementary Figure 3) or daily anomaly over Sep–Dec (Supplementary Figure 4b) as in the observational analyses.

There are 253 individual reforecasts (23 years×11 members) initialized on 01 September. We compared results using reforecast members with that using ensemble means, and find they are similar. We present results using reforecast members here taking advantage of large samples.

### Multiple linear regression

A multiple linear regression method was used to explore the influence of ENSO and IOD on the Rossby wave sources and wave activity flux. The two predictors are SST-Nino3.4 representing ENSO and the SST-DMI representing the IOD. The diagnostic produces two regression patterns: one is associated with influence from ENSO, and the other from IOD.

In Fig. 3c–f the multiple regressions were carried out using Sep–Oct mean anomalies over 1979–2015. Further we scaled the resulting regression patterns with the observed amplitude of SST-DMI (−1.66 °C) and SST-Nino3.4 (−0.78 °C) in 2016 to better reflect respective ENSO and IOD contributions to the Rossby wave sources and wave activity flux for Sep–Oct 2016.

## Electronic supplementary material

Supplementary Information

## Data Availability

The NSIDC sea-ice concentration data set is available from its website (`ftp://sidads.colorado.edu/pub/DATASETS/nsidc0051_gsfc_nasateam_seaice/final-gsfc). The ERA-Interim reanalysis data set is from ECMWF website (http://apps.ecmwf.int/datasets/data/interim-full-moda). NOAA-OIv2 SST data set is available from NOAA web site (https://www.esrl.noaa.gov/psd/data/gridded/data.noaa.oisst.v2.html). HadISST data set is available from the Met Office website (http://www.metoffice.gov.uk/hadobs/hadisst/data/download.html). The monthly mean OLR data set is available from NOAA website (https://www.esrl.noaa.gov/psd/data/gridded/data.interp_OLR.html). The daily and monthly SAM indices are from the NOAA CPC website (http://www.cpc.ncep.noaa.gov/products/precip/CWlink/daily_ao_index/history/method.shtml). The MJO phase data are from the Bureau of Meteorology real-time multivariate MJO (RMM) index (http://www.bom.gov.au/climate/mjo/graphics/rmm.74toRealtime.txt). More information on the MJO phase diagram can be found at http://www.bom.gov.au/climate/mjo/
