# Supplementary material for: Compounding tropical and stratospheric forcing of the record low Antarctic sea-ice in 2016
Source: Nat Commun. 2019 Jan 2;10:13. doi: 10.1038/s41467-018-07689-7 (PMC6312885; doi:10.1038/s41467-018-07689-7)
Supplement: Supplementary file 1 — Supplementary Information [file 41467_2018_7689_MOESM1_ESM.pdf]

Supplementary Information

Compounding Tropical and Stratospheric Forcing of the Record Low Antarctic Sea-Ice in 2016

Guomin Wang[1], Harry H Hendon[1,2], Julie M Arblaster[2,3,4], Eun-Pa Lim[1], S Abhik[3] and Peter van Rensch[3]

[1] *Bureau of Meteorology, Melbourne, Victoria, Australia*

[2] *ARC Centre of Excellence for Climate Extremes, Australia*

[3] *School of Earth, Atmosphere and Environment, Monash University, Clayton, Victoria, Australia*

[4] *National Center for Atmospheric Research, Boulder, Colorado, USA*

Correspondence and request for materials should be addressed to G.W. (g.wang@bom.gov.au)

Table of content:

Supplementary figures 1-7.

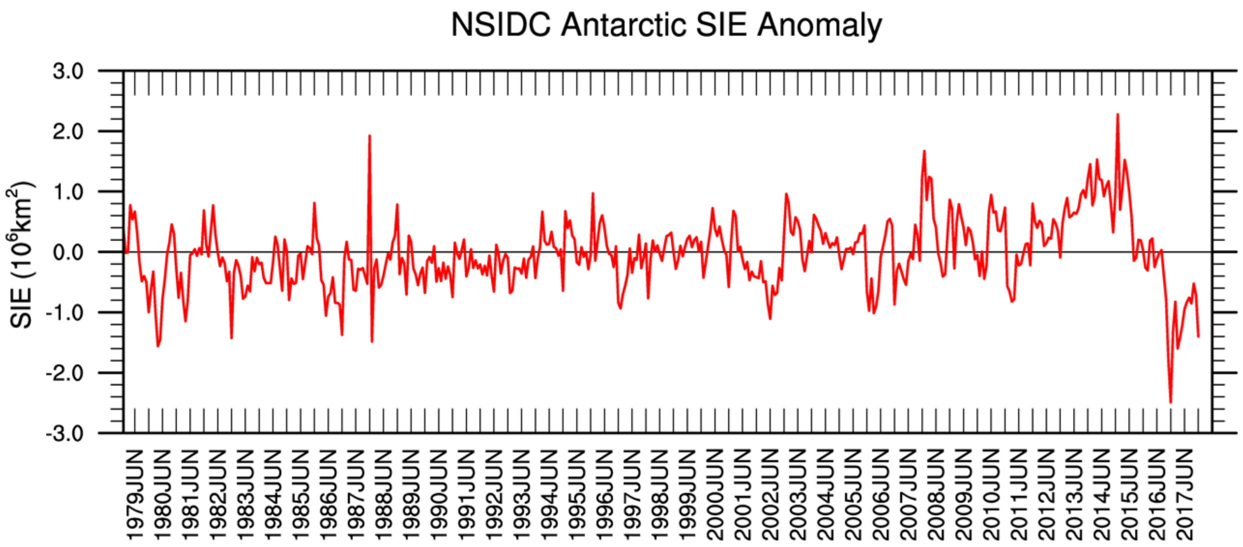

Supplementary Figure 1 | Monthly time series of the NSIDC NASA Team Antarctic sea-ice extent anomalies from January 1979 to December 2017. The anomalies are relative to 1979-2015 mean, unit 10^6 km^2. The ticks along x-axis are for June (labelled) and December (without label) each year. Plots were generated using the NCAR Command Language version 6.4.0 (www.ncl.ucar.edu).

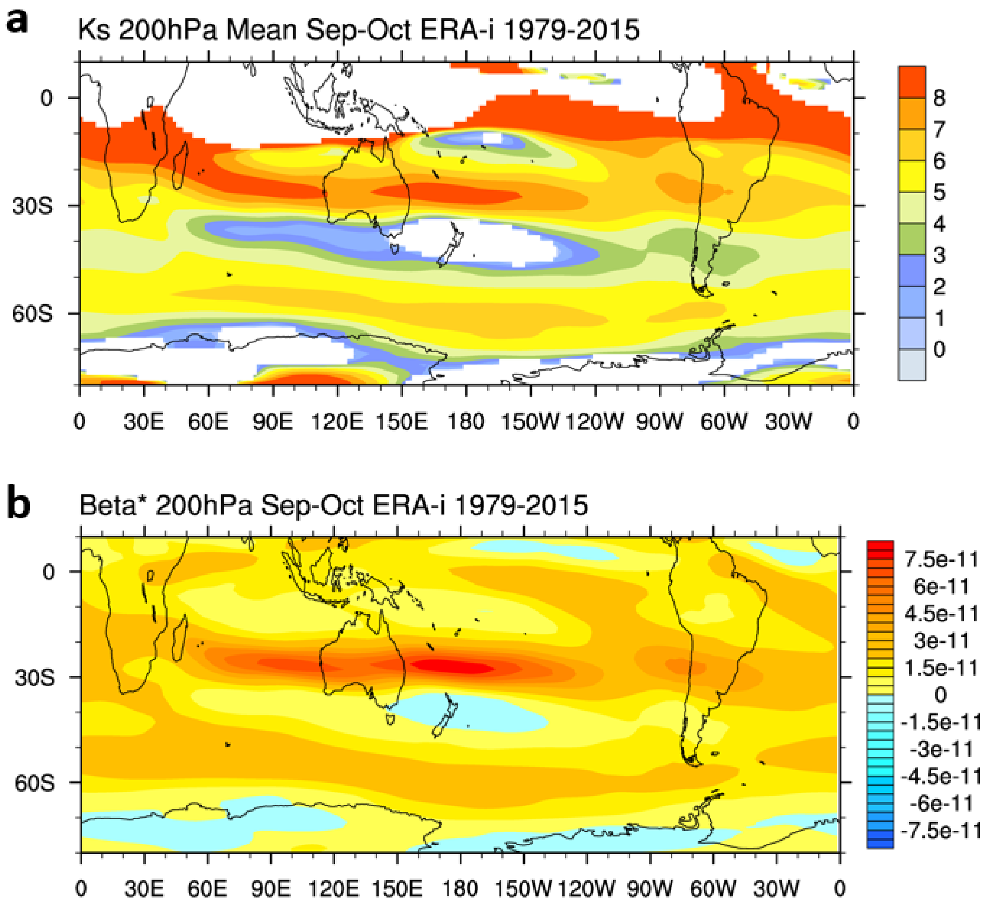

Supplementary Figure 2 | Total stationary Rossby wavenumber and meridional gradient of the mean absolute vorticity for mean conditions Sep-Oct 1979-2015 at 200 hPa . a, total stationary Rossby wavenumber (dimensionless unit; see Methods). **b**, meridional gradient of the mean absolute vorticity **(**m^{-1} s^{-1}**).** The white areas in **a** indicate where the total wavenumber is imaginary due to either negative meridional gradient of vorticity (e.g., surrounding New Zealand) or in regions where mean zonal winds is easterly (e.g., the tropics). See Methods for further details. Plots were generated using the NCAR Command Language version 6.4.0 (www.ncl.ucar.edu).

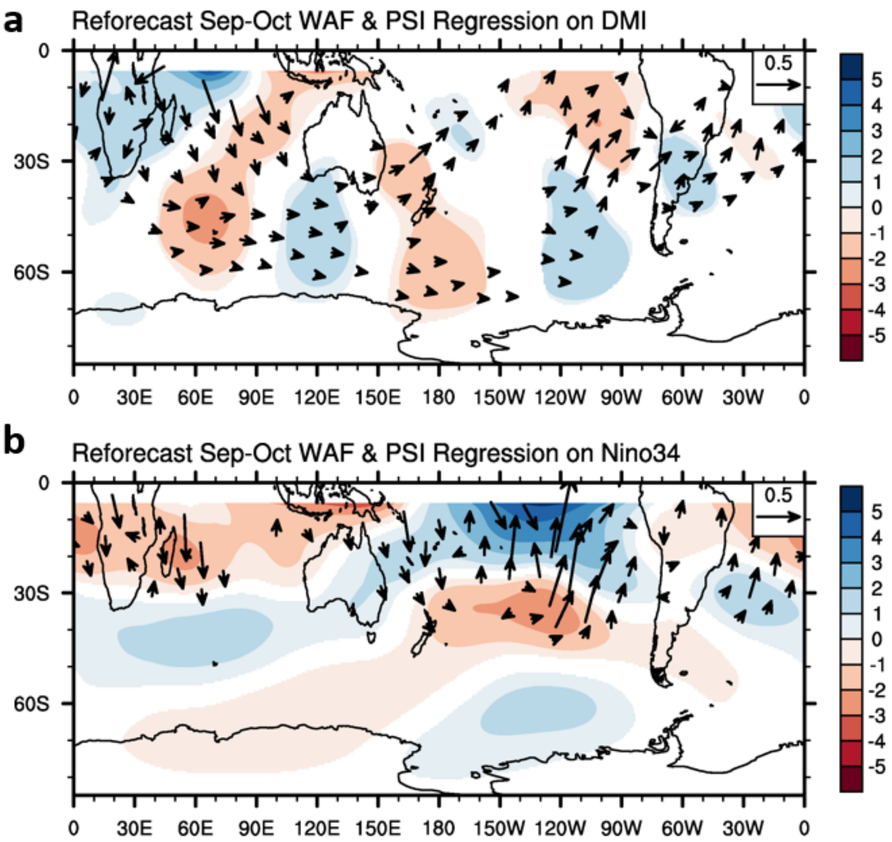

Supplementary Figure 3 | Simulated Rossby wave trains and wave activity flux during September-October based on reforecasts using coupled model seasonal prediction system 1990-2012. a, partial regression of eddy streamfunction (shading; interval $1x10^6$ m^2s) and wave activity flux (WAF, vectors, m^2s^{-2}) at 200 hPa onto SST-DMI. **b,** same as **a** but all fields are partial regressions onto SST-Nino3.4. The reforecasts consist of an 11-member ensemble that was initialized from observed atmosphere-ocean states for each 1 September 1990-2012 (see Methods). The regressions were performed using the predicted SST-DMI and Nino34 indices, but for plotting the coefficients are scaled by the observed magnitude of the SST-DMI and Nino34 indices for Sep-Oct 2016. Regression coefficients are shown only when p<0.10 using a two-sided *t*-test. For clarity, WAF vectors with magnitude less than 0.1 m^2s^{-2} are masked. In Southern Hemisphere, the negative streamfunction anomaly corresponds to positive geopotential height anomaly and thus is coloured in reds; similarly, positive streamfunction anomaly is coloured in blues. Plots were generated using the NCAR Command Language version 6.4.0 (www.ncl.ucar.edu).

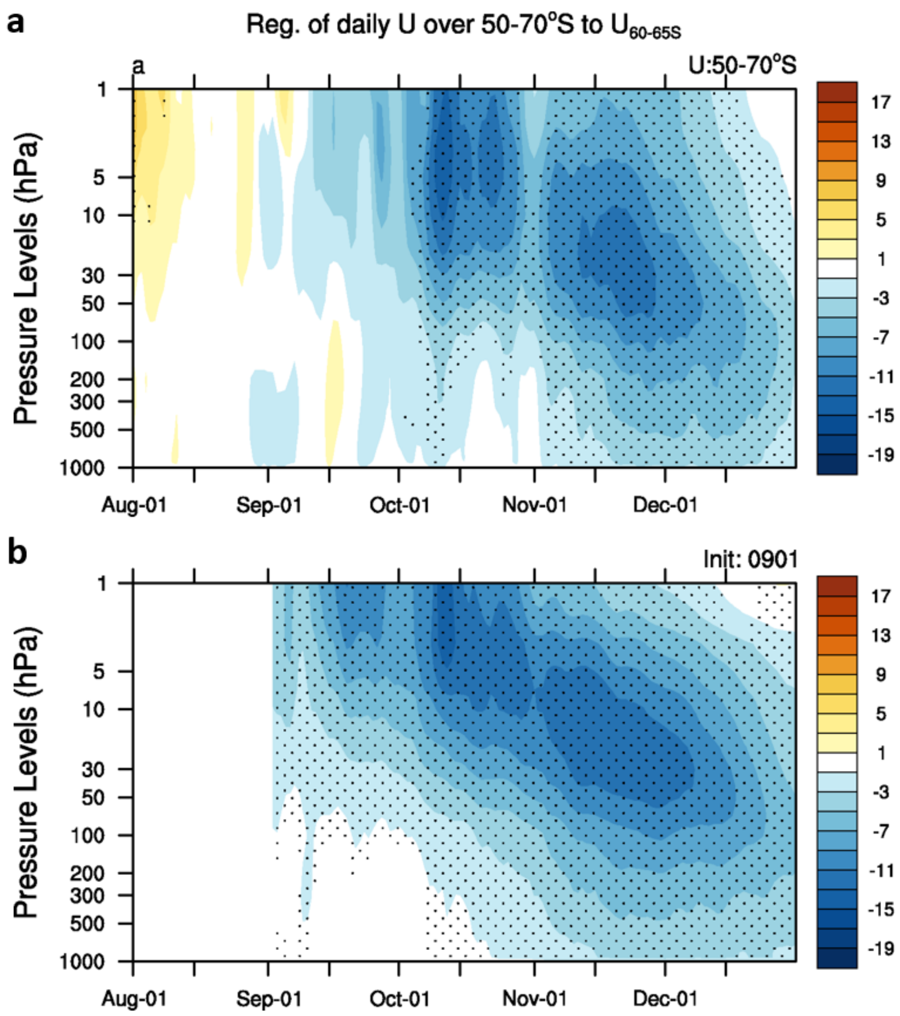

Supplementary Figure 4 | Historical lagged relationship between November-December surface zonal wind index $U_{60\text{-}65S}$ and stratospheric polar vortex. a, Lagged regression of zonal mean zonal wind (averaged over 50º-70ºS) as a function of pressure onto surface November-December zonal wind index $U_{60\text{-}65S}$ using ERA-interim over 1979-2015. **b**, same as **a** but the regression was computed using seasonal prediction reforecasts initialized on the 1st of September during 1990-2012 (see Methods). For display, the regression coefficients have been scaled by the observed value of $U_{60\text{-}65S}$ during Nov-Dec 2016 (as shown in Fig. 1f) to estimate the expected magnitude of wind anomalies that should have resulted in 2016. Stippling indicates the statistical significance at the 5% level, estimated with a two-sided *t*-test. Plots were generated using the NCAR Command Language version 6.4.0 (www.ncl.ucar.edu).

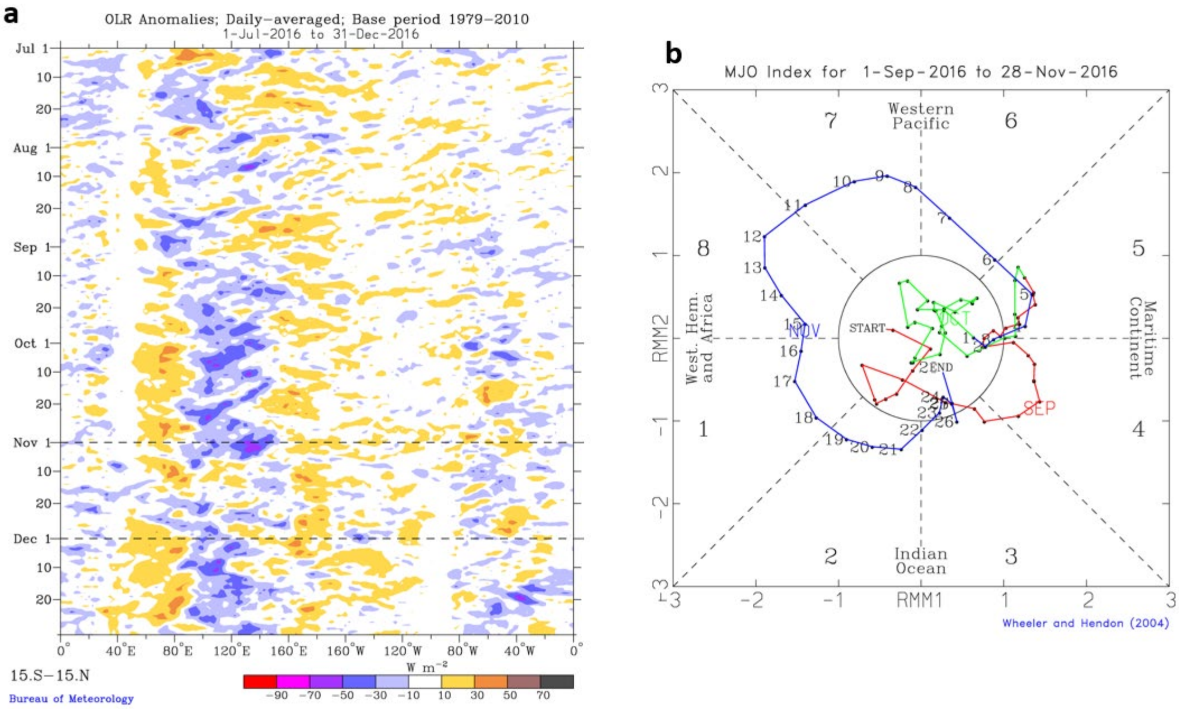

Supplementary Figure 5 | Behavior of Madden-Julian Oscillation (MJO) during 2016. a, Daily Hovmöller plot of OLR anomalies averaged 15°S-15°N during July-December 2016**.** The dates 1 November and 1 December are indicated by dashed horizontal lines**. b,** MJO phase diagram during September-November 2016. The portion during November is blue coloured with day of month labelled. The MJO is considered strong when its amplitude is outside of the unit circle[1]. The MJO's propagation from west to east is indicated by an anti-clockwise rotation with time (e.g. watching day numbers during November). Enhanced convection is indicated by blue shading in **a**. The persistent enhanced convection in eastern Indian Ocean associated with negative IOD during Sep-Oct 2016 is seen to be disrupted by passage of the MJO during first half of Nov 2016. Plots were sourced from the Bureau of Meteorology website (http://www.bom.gov.au/climate/mjo/).

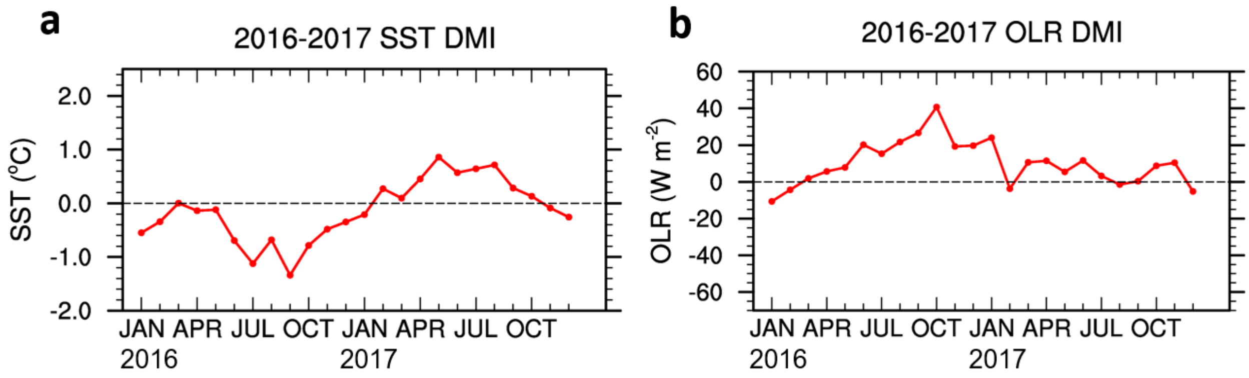

Supplementary Figure 6 | Time series of monthly anomalies during January 2016 to December 2017. a, SST-DMI, °C; **b,** OLR-DMI, W/m^2**.** The anomalies are relative to 1979-2015 mean. Plots were generated using the NCAR Command Language version 6.4.0 (www.ncl.ucar.edu).

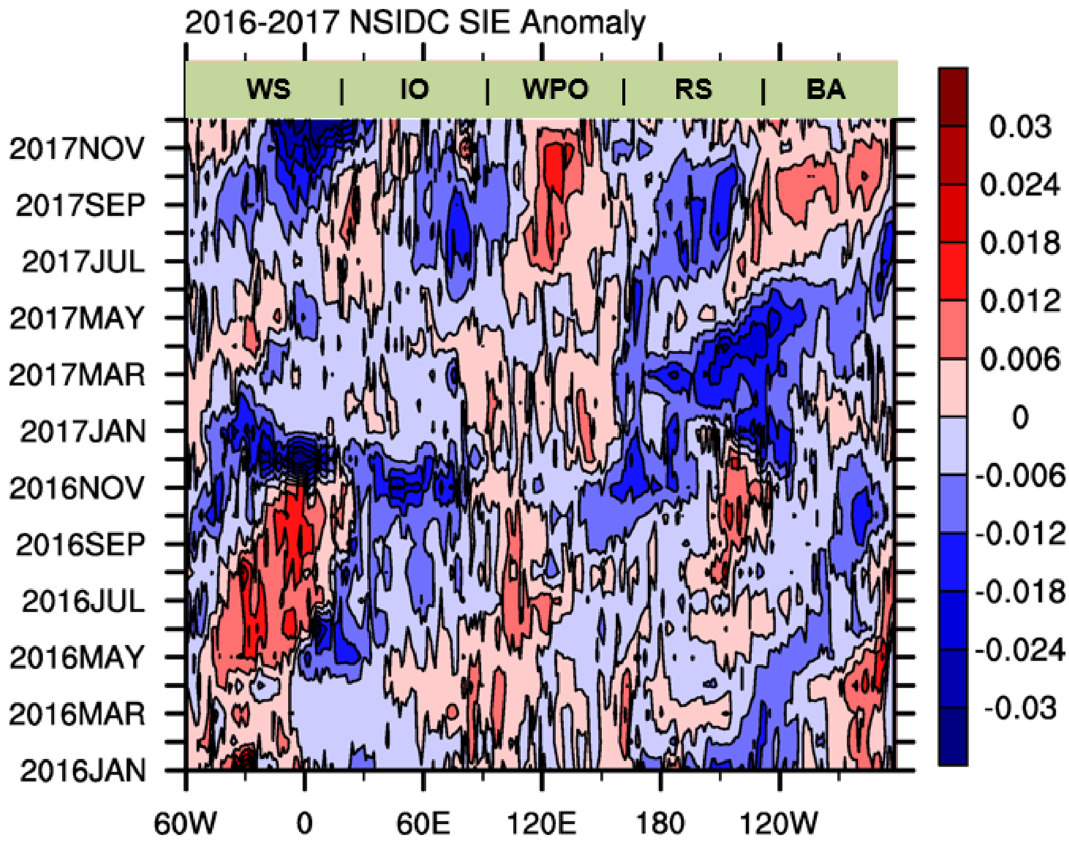

Supplementary Figure 7 | Monthly Hovmöller plot of the Antarctic sea-ice extent anomalies for January 2016 to December 2017. Longitude starts from 60°W for matching Antarctic sectors shown on the top: WS, Weddell Sea (60°W-20°E); IO, Indian Ocean (20°E-90°E); WPO, Western Pacific Ocean (90°E-160°E); RS, Ross Sea (160°E-130°W); BA, Bellingshausen and Amundsen Seas (130°W-60°W). The anomalies are relative to 1979-2015 means. Contour interval $6x10^3$ km^2**.** Plots were generated using the NCAR Command Language version 6.4.0 (www.ncl.ucar.edu).

References

1. Wheeler, M. C. & Hendon, H. H. An All-Season Real-Time Multivariate MJO Index: Development of an Index for Monitoring and Prediction. *Mon. Weather Rev.* **132,** 1917–1932 (2004).
